# Polyethylenimine-mediated expression of transgenes in the acinar cells of rats salivary glands *in vivo*

**DOI:** 10.3389/fcell.2014.00074

**Published:** 2015-01-09

**Authors:** Monika Sramkova, Laura Parente, Timothy Wigand, Myo-Pale' Aye, Akiko Shitara, Roberto Weigert

**Affiliations:** Intracellular Membrane Trafficking Unit, Oral and Pharyngeal Cancer Branch, National Institute of Dental and Craniofacial Research, National Institutes of HealthBethesda, MD, USA

**Keywords:** intravital microscopy, *in vivo* transfection, polyethyleneimine, aquaporin 5, non-viral gene delivery, salivary glands, acinar cells

## Abstract

Non viral-mediated transfection of plasmid DNA provides a fast and reliable way to express various transgenes in selected cell populations in live animals. Here, we show an improvement of a previously published method that is based on injecting plasmid DNA into the ductal system of the salivary glands in live rats. Specifically, using complexes between plasmid DNA and polyethyleneimine (PEI) we show that the expression of the transgenes is directed selectively to the salivary acinar cells. PEI does not affect the ability of cells to undergo regulated exocytosis, which was one of the main drawbacks of the previous methods. Moreover PEI does not affect the proper localization and targeting of transfected proteins, as shown for the apical plasma membrane water channel aquaporin 5 (AQP5). Overall, this approach, coupled with the use of intravital microscopy, permits to conduct localization and functional studies under physiological conditions, in a rapid, reliable, and affordable fashion.

## Introduction

Intravital microscopy (IVM) has enabled researchers to image several biological processes in live multicellular organisms and in real time (Pittet and Weissleder, 2011; Ritsma et al., 2012; Weigert et al., 2013). In the last decade, this approach has permitted to image subcellular organelles in live rodents with temporal and spatial resolution almost identical to that achieved in cell cultures (Dunn et al., 2002; Weigert et al., 2013; Weigert, 2014). This has been possible by the development of procedures aimed at minimizing the motion artifacts due to the heartbeat and respiration, which otherwise would make imaging at a submicron scale an impossible task. In particular, we have developed a rodent salivary gland model that has enabled (1) tracking individual structures for extended periods of time, and (2) extracting quantitative data in a rigorous fashion. This approach has been used to study various aspects of the dynamics of endocytosis and regulated exocytosis in the salivary epithelium of both rats and mice (Masedunskas and Weigert, 2008; Masedunskas et al., 2011a, 2012), and recently, it has revealed a novel modality of mitochondrial metabolism that cannot be observed in *in vitro* models (Porat-Shliom et al., 2014).

So far, pharmacological tools have been the main approach to study the molecular mechanisms regulating the dynamics of subcellular events in live animals (Sandoval et al., 2004; Masedunskas et al., 2011a). One of the current challenges is to generate molecular tools that can be rapidly expressed *in vivo*, and interfere with the process of interest. An elegant approach is to develop transgenic mice engineered to express fluorescently tagged molecules, which act as reporters for the pathway of interest, and, at the same time, lack or express the specific molecule under investigation. Although the application of gene editing techniques to mice (e.g., zinc finger or CRISPR is rapidly growing (Sung et al., 2012; Yang et al., 2013), these methods are still expensive and time consuming. Viable alternatives are *in vivo* transient transfection of transgenes and/or shRNAs, which can be accomplished by either viral or non-viral mediated delivery (Boulaiz et al., 2005; Kawakami et al., 2008; Wang et al., 2013). Viral-mediated gene transfer is the most widely used approach since it allows to transfect a high number of cells and to efficiently deliver and express the transgenes in non-dividing or slowly dividing cells (Boulaiz et al., 2005). This approach provides with the opportunity to perform biochemical analysis on the transfected tissues. In small animals, the main viral vectors currently used are: recombinant adenovirus serotype 5 (rAD5), adeno-associated virus serotype 2 (AAV2), and lenti viruses (Snyder and Francis, 2005; Adriaansen et al., 2010; Wilson et al., 2013). However, viral-mediated transfections have some limitations, which include adverse reactions due to the host immune response (Sun et al., 2003). Moreover, the generation of viruses is time consuming and subjected to safety restrictions. To overcome these issues, non viral-mediated techniques have been attempted in various organs since the early 90's, and in particular, direct injections of plasmid DNA in rodent skeletal muscle have proven to be quite successful (Wolff et al., 1992). Following up on early studies in SGs (Goldfine et al., 1997), we have developed and characterized a transfection method that can be used with commercially available plasmids, and thus suitable for rapid screenings and mutation analysis. We have shown that plasmid DNA can be transfected in the SGs of live rats using three different modalities of administration, which ensure to direct the expression of the transgene to selected cell populations (Sramkova et al., 2009, 2012). Although the yield of non viral-based transfections is an order of magnitude lower than viral-based methods, this approach is robust and enables performing single cell imaging *in vivo*. Here we show a variation of one of these transfection methods that is based on the use of polyethyleneimine (PEI) a molecule previously used as a vehicle to compact and deliver siRNA *in vivo* (Kircheis et al., 2001; Nimesh, 2012). This approach permits the selective transfection of acinar cells in the SG epithelium in a rapid and reliable fashion without affecting the properties and the localization of the transgenes.

## Results and discussion

### PEI facilitates the targeting of the plasmid DNA to the acinar cells of the salivary glands *in vivo*

**Figure 1:**
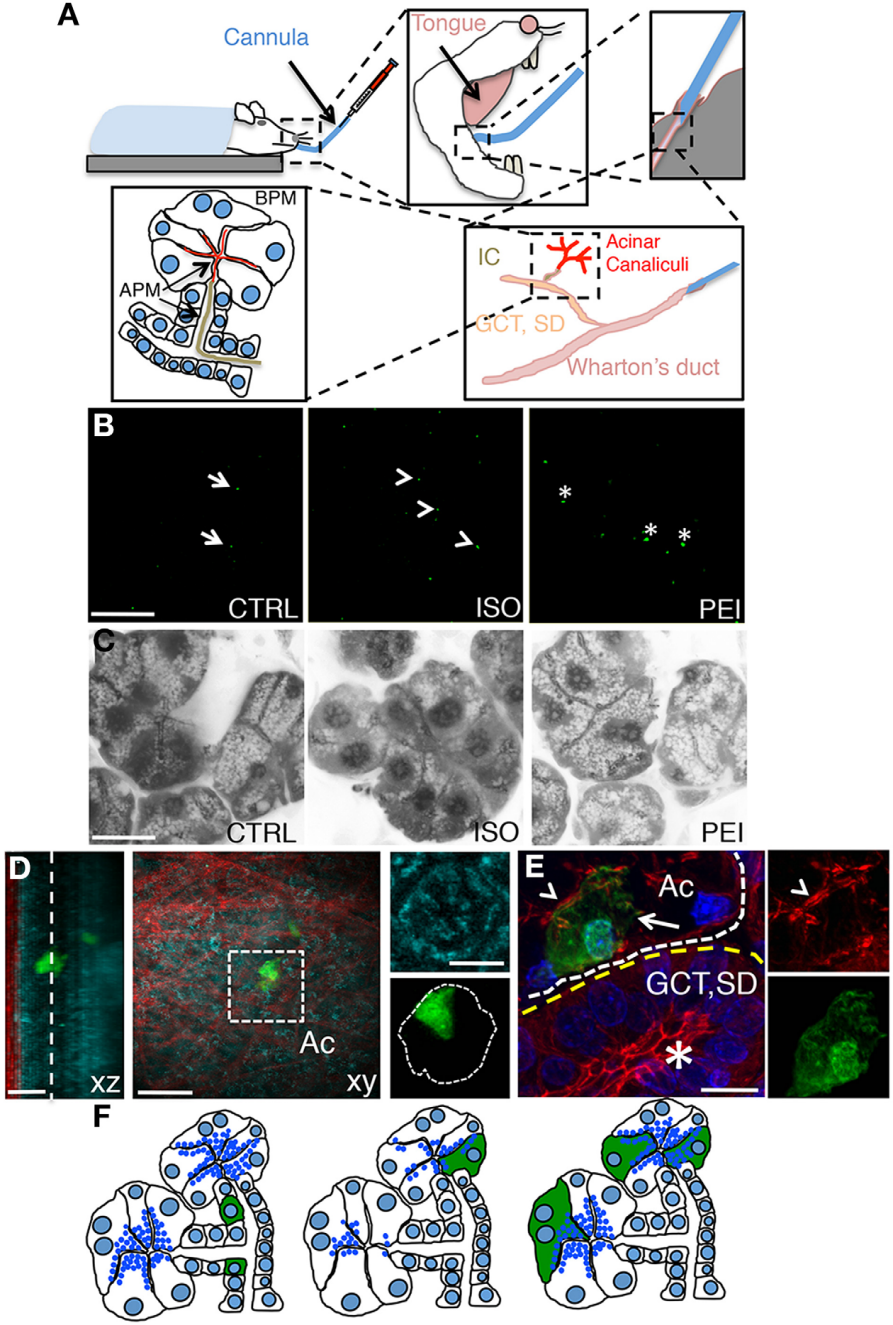
**Non viral-mediated expression of transgenes in rat salivary glands**. **(A)** Diagram of the transfection procedures in anesthetized rats. A thin polyethylene cannula is inserted into the Wharton's duct, as described in the Methods sections. The plasmid DNA is gently injected and diffuses into the large ducts (granular convoluted tubules or GCT, and the striated ducts or SD), then into the intercalated ducts (IC), and finally into the acinar canaliculi. Plasmid DNA accesses the epithelium through the apical plasma membrane (APM, arrows). **(B)** *In vivo* transfection with plasmid DNA -12 μg of Plasmid DNA encoding for pVenus were injected alone (CTRL, left panel), after SC injection of isoproterenol (ISO, center panel), or mixed with PEI as described in the Methods (PEI, right panel). After 16 h the submandibular SGs were excised and immediately imaged by two-photon microscopy using a 10X objective (excitation 930 nm) to reveal the cells expressing pVenus (arrows). Scale bar, 1 mm. **(C)** Effect of PEI on regulated exocytosis. Mice expressing cytoplasmic GFP were treated as described in B and the SGs imaged by confocal microscopy (excitation 488 nm) to estimate exocytosis of the secretory granules, as previously reported (Milberg et al., 2013). In the inset the apical plasma membrane (arrows) and the secretory granules (arrowheads) are highlighted. Scale bar, 20 μm. **(D–E)** PEI-mediated transfection in SGs drives expression into acinar cells. **(D)** Excised glands transfected with pVenus/PEI were imaged by two-photon microscopy using a 60x water immersion objective **(D)** or processed for immunofluorescence and imaged by confocal microscopy **(E)**. **(D)** Two Z-stacks from the same area of the sample were acquired twice by using two different excitation wavelengths, respectively: 740 nm to reveal the epithelium of the gland (cyan) and 930 nm to reveal pVenus (green) and collagen fibers (red). The Z-stacks were combined and shown as xz (left) or xy (center) projection. The cell expressing pVenus is part of an acinus (inset, right). Scale bar, 20 μm. **(E)** The SGs were labeled for phallodin (red) and the nuclear staining Hoechst (blue). The cell expressing pVenus (green) is part of an acinus (white broken line) as identified by the actin-labeled acinar canaliculi (arrowhead). Large ducts (yellow broken line), as identified by the characteristic actin pattern (asterisk) did not express any pVenus. Scale bar, 10 μm. **(F)** Diagram summarizing the pattern of expression of plasmid DNA in SGs. pVenus was expressed in cells of the IC under control conditions (left) and in acinar cells either upon stimulation with ISO (center) or administration with PEI (right).

**Figure 2:**
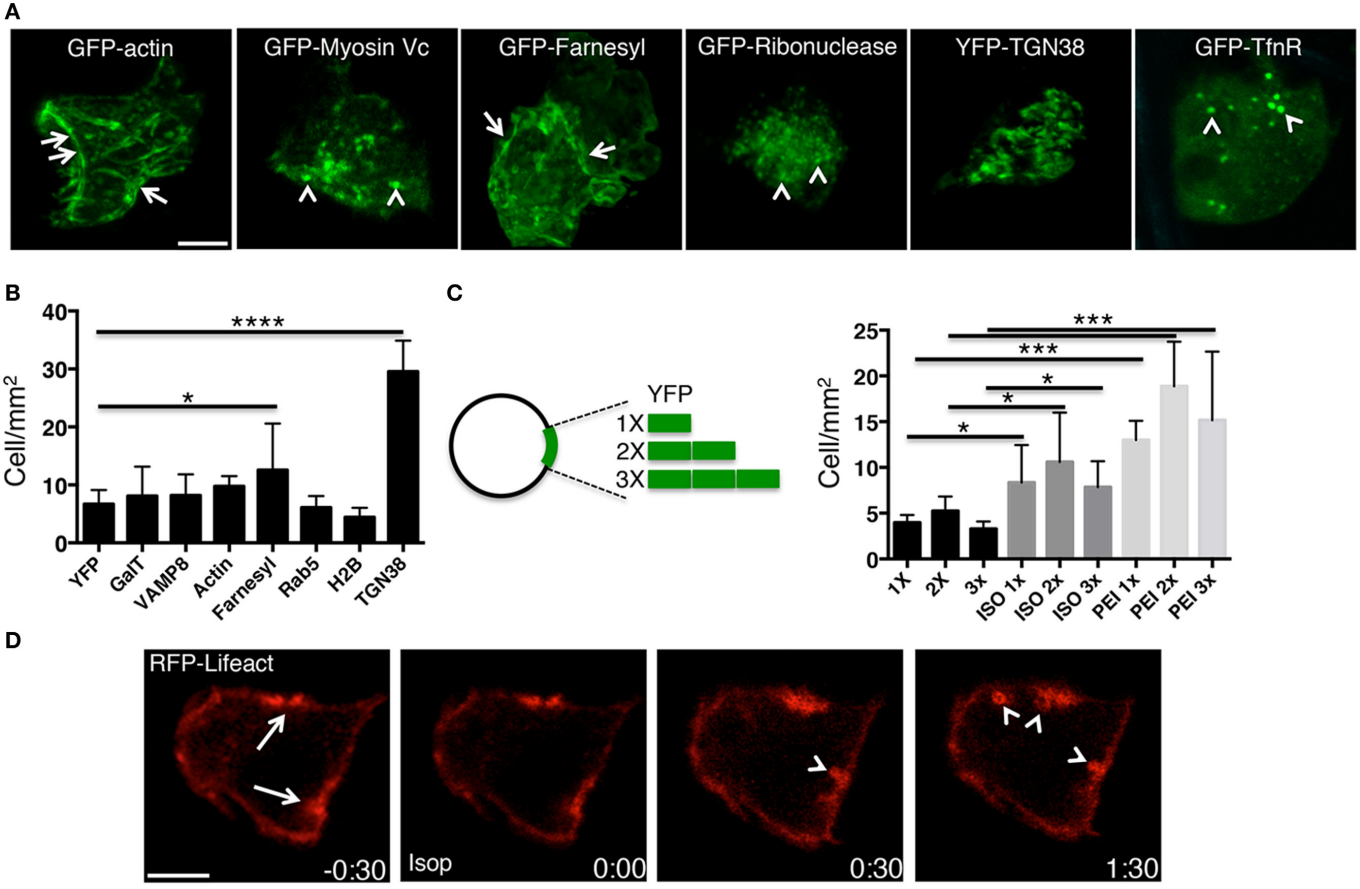
**PEI enables the expression of several transgenes in the acinar cells of the SGs**. **(A–C)** Plasmid DNAs encoding either for selected fluorescently tagged proteins **(A,B)** or for multiple copies of pVenus **(C)** were injected into the SGs of anesthetized rats under different conditions. **(A)** High magnification images acquired by two-photon microscopy (excitation 930 nm, 60x water-immersion objective) to reveal the intracellular localization of selected transfected transgenes. **(B–C)** The efficiency of expression of the transgene was determined as described in the Methods and expressed as cells/mm2. Values represent average ±S.D. (*N* = 3). Statistical significance was calculated by using a *t*-test ^*^p < 0.1 ^***^p < 0.001 ^****^P < 0.0001. **(B)** Plasmids encoding for various cellular proteins and mixed with PEI are shown. **(C)** Plasmids containing 1 (1X), 2 (2X) or 3 (3X) copies of pVenus fused together where injected into the salivary duct alone, after stimulation with ISO, or mixed with PEI. **(D)** Plasmid DNA encoding for the F-actin reporter RFP-lifeact was mixed with PEI and injected into the salivary duct of anesthetized rats. After 24 h the SGs were exposed and imaged by confocal microscopy (excitation 561 nm). In acinar cells, RFP-life act was loclaized at the plasma membrane and in particular at the APM (arrows). SC injection of isoproterenol (time 0:00) elicited regulated exocytosis. As previously shown (Masedunskas et al., 2011a), secretory granules fused with the APM and recruited a F-actin coat (arrowheads). Scale bar, 10 μm.

We have previously shown that plasmid DNA can be delivered to the submandibular SGs in live rats by retro-injection trough the salivary duct (or Wharton's duct). Plasmid DNA is loaded into the ductal system and the acinar canaliculi, thus gaining access to the salivary epithelium from the apical plasma membrane (APM) (Figure 1A). Specifically, we have shown that although plasmid DNA is uptaken by all the cells in the epithelium, only few of the proliferating cells of the intercalated ducts express the transgenes (Sramkova et al., 2012) (Figures 1B,F). On the other hand, plasmid DNA mixed with empty rAD5 particles elicited the expression of the transgenes primarily in large ducts (Sramkova et al., 2009). Finally, we have shown that plasmid DNA is targeted exclusively to the acinar cells during β adrenergic-mediated stimulation of regulated exocytosis (Sramkova et al., 2009). Indeed, during secretion, plasmid DNA is internalized into the acinar cells by virtue of compensatory endocytosis that is elicited to maintain the homeostasis of the APM (Sramkova et al., 2009; Masedunskas et al., 2011b) (Figures 1B,F). However, one of the main drawbacks of this approach is the fact that β-adrenergic stimulation induces an extensive degranulation of the acinar cells (Masedunskas et al., 2011a) (Figure 1C, central panel). Since the biogenesis of the secretory granules is a slow process, their full complement is reconstituted in 24–36 h, which is beyond the optimal expression of the transgene (16–24 from the administration of the plasmid). Therefore, this method cannot not be used to study processes such as regulated exocytosis, and trafficking from the TGN, where the granules are originated. To overcome this issue we sought to establish a method that could enhance the low level of endocytosis that occurs at the APM of the acinar cells without inducing exocytosis (Sramkova et al., 2012). Specifically, we tested PEI, which has been extensively used to deliver siRNAs both *in vitro* and *in vivo*. Although its precise mechanisms of action is not fully understood, PEI has been suggested to: (1) facilitate the packing of nucleotide strands, (2) increase the endocytosis of the complex PEI-siRNA, and (3) promote endosomal rupture, thus releasing the complex into the cytoplasm (Neu et al., 2005; Creusat et al., 2010). PEI was mixed with plasmid DNA encoding for the monomeric YFP (see Materials and Methods) and injected through the Wharton's duct of anesthetized rats. After 16 h the animals were anesthetized and the SGs were exposed and imaged by intravital two-photon microscopy (Figure 1B). Using a low magnification objective we sampled large areas of the tissue and observed that ≈10 cells/mm^2^ expressed the transgene, a level comparable to that observed in β adrenergic- stimulated rats (Figure 2B). To determine whether PEI stimulated regulated exocytosis we used mice expressing cytoplasmic GFP that enable the visualization of the secretory granules (Masedunskas et al., 2011a; Milberg et al., 2013). Notably, SC injection of isoproterenol elicited robust exocytosis whereas PEI did not (Figure 1C and insets). The yield of transfection was maximal between 16–24 h from the administration (not shown). Moreover, we found that the transgene was expressed in acinar cells, as shown by performing either two-photon microscopy in freshly excised glands (Figure 1D) or immunocytochemistry and confocal microscopy (Figure 1E).

PEI facilitates the expression of molecules other than YFP, as shown by administering plasmid DNAs encoding for various molecules, such as cytoplasmic (e.g., actin, myosin Vc), nuclear (e.g., H2B), membrane-associated (e.g., Rab5), single and multiple spanning transmembrane proteins (e.g., TGN38, TfnR, VAMP8) (Figures 2A,B). The efficiency of transgene expression did not correlate with any characteristics of the expressed proteins or the size of the plasmid, as shown by expressing a transgene generated by fusing one, two or three copies of YFP (Figure 2C).

Our data show that PEI is an effective agent in facilitating the delivery and expression of transgenes into the acinar cells of rat salivary glands *in vivo*. Moreover, PEI does not cause any obvious cellular toxicity, as shown by the fact that regulated exocytosis and the dynamics of assembly of the actin cytoskeleton were not affected (Figure 2D). Finally, we did not observe any general signs of toxicity in the whole animal, at least within 24–48 h from the administration of PEI (not shown).

### PEI does not affect protein targeting to specialized domains of the plasma membrane

Next, we checked whether PEI affected the proper targeting of plasma membrane proteins to different domains of the acinar cells in the live animal. To this end, we investigated the expression and the localization of aquaporin 5 (AQP5), a water channel that regulates fluid secretion in mammalian SGs and is targeted to the APM via a sorting signal encoded in its C-terminus (Raina et al., 1995; Wellner et al., 2005; Delporte and Steinfeld, 2006; Horsefield et al., 2008).

**Figure 3:**
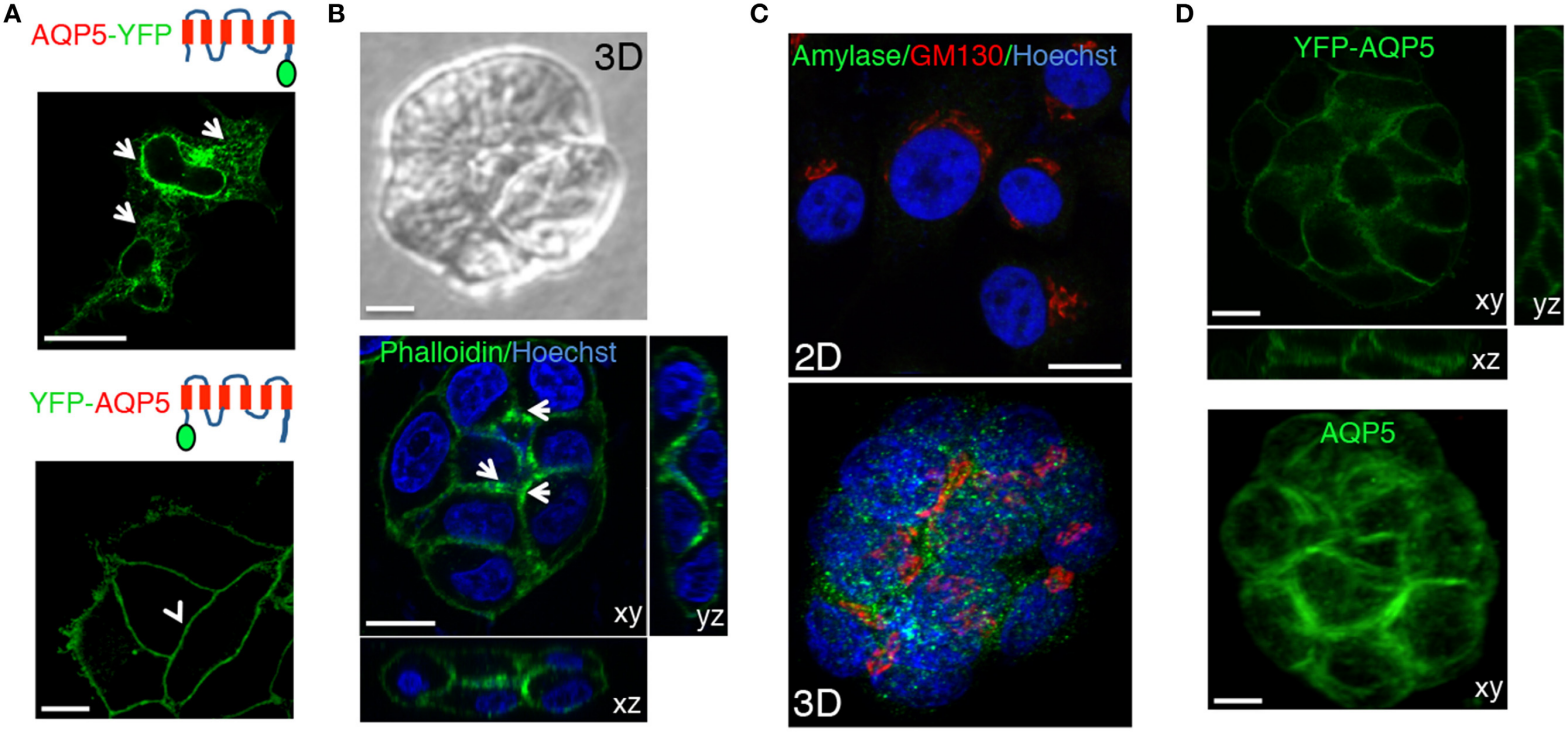
**Characterization of the fluorescently tagged AQP5**. **(A)** Human AQP5 was tagged either at the **(C)** or the N-terminus and transiently expressed in HSG cells grown on glass slides. AQP5-YFP is localized in the ER and the nuclear envelope (arrows), whereas YFP-AQP5 is localized at the APM (arrowheads). **(B–D)** HSG cells (**B**, **C**, and **D** lower panel) or HSG cells stably expressing YFP-AQP5 (**D**, upper panel) were grown for 96 h in matrigel to form acinar-like structures or on glass coverslips (**C**, upper panel) and imaged by either phase contrast (**B**, upper panel) or confocal microscopy (**B**, **C**, lower panel and **C**) in order to acquire Z-stacks. Cells were fixed and processed to reveal F-actin and the nuclei (**B**, lower panel), YFP-AQP5 (**C**, upper panel), endogenous AQP5 (**C**, lower panel), amylase **(C)** and the Golgi apparatus (GM130, **C**). Maximal projections of the xy view and side views (xz, yz) show that F-actin is partially polarized in the acinar-like cells (arrows) whereas both endogenous and ectopically expressed AQP5 did not. Scale bars, 5 μm.

Trafficking, localization, and targeting mechanism of AQP5 have been primarily investigated in cell cultures grown on solid substrates (Tada et al., 1999), in polarized MDCK cells (Nejsum and Nelson, 2007; Karabasil et al., 2009), and only to a small extent in native tissue in rodents (Gresz et al., 2004; Wellner et al., 2005; Matsuzaki et al., 2006). For these studies AQP5 has been tagged with fluorescent proteins (e.g., GFP or RFP) and the behavior of these constructs has been shown to be dependent on the experimental system and the position of the tag. We began by characterizing various constructs tagged either at the C- or the N-terminus (both human and rat AQP5) in cultured salivary glands-derived HSG cells (Royce et al., 1993). We used the monomeric form of the yellow fluorescent proteins (YFP) that has better quantum efficiency than the GFP when excited by 2-photon microscopy (Nagai et al., 2002). In HSG cells, we observed that AQP5-YFP was expressed but did not get exported out of the endoplasmic reticulum (Figure 3A, upper panel, arrows). On the other hand, YFP-AQP5 was properly targeted to the PM as previously reported (Wellner et al., 2005), and no intracellular vesicles were detected (Figure 3A, lower panel, arrowhead). Next, we generated stable HSG cells expressing YFP-AQP5 grown either as a confluent monolayer or in matrigel (Hoffman et al., 1996, 1998). In matrigel, HSG cells form acinar-like structures, which exhibit a partial polarization, as shown by phalloidin labeling (Figure 3B) and expression of amylase, a marker for acinar differentiation (Figure 3C) (Hoffman et al., 1996). YFP-AQP5 was properly targeted to the plasma membrane both in monolayers and in matrigel (Figure 3D). However, in acinar-like structures AQP5 did not exhibit any polarized localization, suggesting that other components may be required for the proper sorting, such as the μ1-b subunit of the AP1 adaptor complex (Figure 3D) (Ohno et al., 1999). Similar results were obtained expressing rat YFP-AQP5 (not shown).

**Figure 4:**
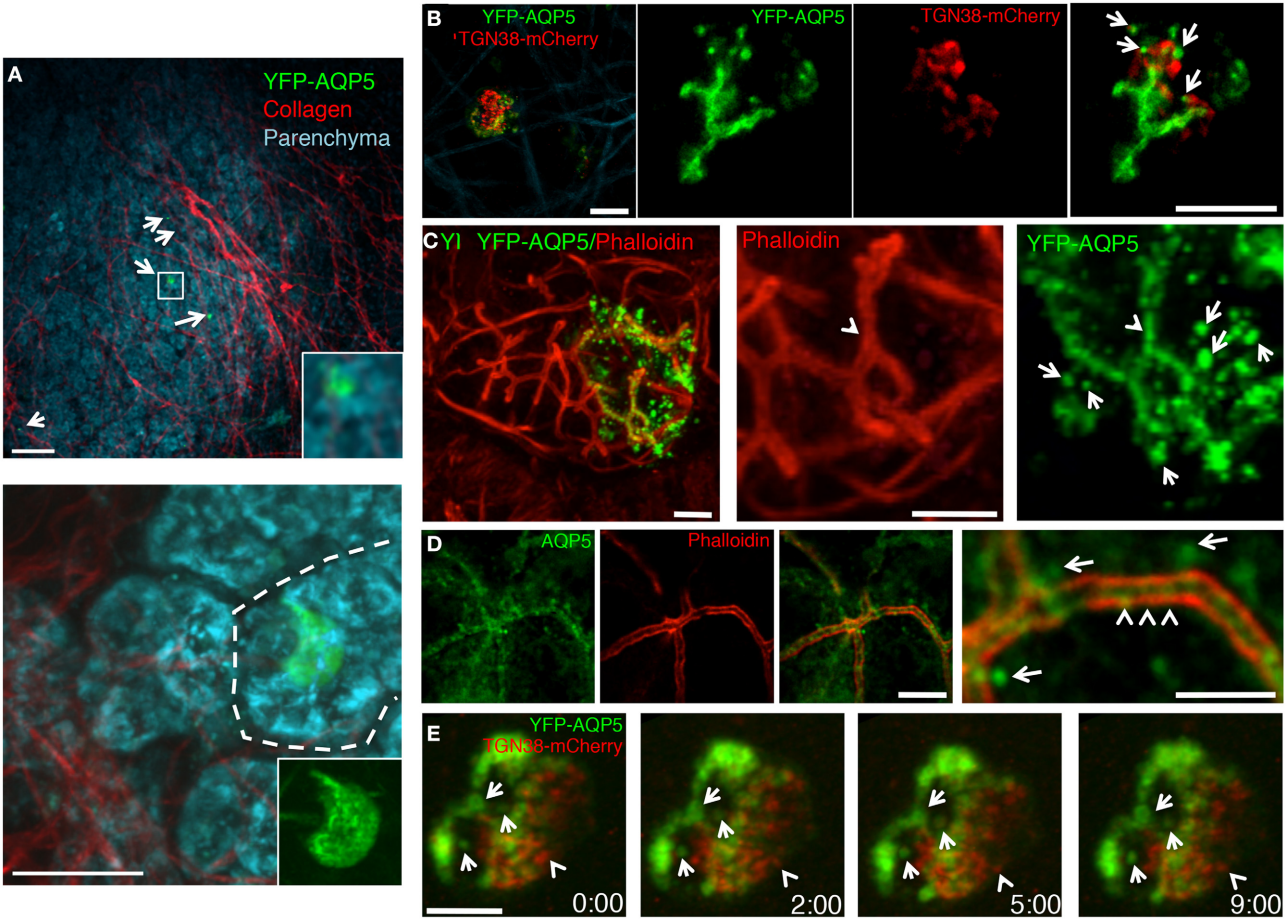
**Targeting of ectopically expressed AQP5 in the SG *in vivo***. The SGs of anesthetized rats were left untreated **(D)** or injected with a plasmid encoding for rat YFP-AQP5 either alone **(A,B)** or in combination with TGN38-mcherry, a marker for the Trans-Golgi Network **(C,E)**. The salivary glands were exposed and imaged by two-photon microscopy **(A,B,E)** or fixed, processed for immunofluorescence and imaged by confocal microscopy **(C,D)**. **(A)** Two Z-stacks from the same area of the sample were acquired twice by using two different excitation wavelengths, respectively: 740 nm to reveal the epithelium of the gland (cyan) and 930 nm to reveal the YFP (green) and collagen fibers (red). The Z-stacks were combined as described in the Methods and shown as maximal projections. Upper panel shows a low magnification of the parenchyma (20X objective) with 5 cells expressing YFP-AQP5 (arrows). Lower panel show an acinus (broken line) with a transfected cell (60X objective). Scale bars, 40 μm. **(B)** Maximal projection of an individual acinar cells (Left panel) and high magnification of a single optical slice (60X objective) showing that YFP-AQP5 (green) is localized a at the APM and in small vesicles (arrows). Scale bars, 5 μm. **(C,D)** Cryosections from SGs were labeled with Rhodamine-Phalloidin (red) alone **(C)**, or with an antibody directed against rat AQP5 (**D**, green). Both ectopically expressed and endogenous AQP5 are localized at the APM (arrowheads) and in small vesicles (arrows). Scale bars, 5 μm. **(E)** Time lapse imaging of an individual acinar cells show that YFP-AQP5-containing vesicles are stationary over a long period of time. Note a TGN38-containng vesicle that pinches off the TGN (arrowhead). Scale bar 3 μm.

Next, we injected the plasmid encoding for rat YFP-AQP5 into the salivary ducts of anesthetized rats by using PEI as vehicle. As expected, YFP-AQP5 was expressed in the SGs (Figure 4A, arrows), and primarily in acinar cells that were identified by endogenous fluorescence emissions (excitation 740 nm), which highlight the cellular architecture of the salivary epithelium (Masedunskas and Weigert, 2008) (Figure 4A, lower panel). YFP-AQP5 was properly targeted to the APM as shown by two-photon microscopy (Figure 4B) and indirect immunofluorescence (Figure 4C, arrows). Interestingly, we observed that YFP-AQP5 was localized onto a series of intracellular vesicles localized in the sub-apical area of the acinar cells (Figures 4B,C, arrows) differently from what observed in cultured cells. These vesicles were not due to the fixation and the processing of the glands since they could be observed in the live animal (Figure 4B) and they were not an artifact of the ectopic expression of YFP-AQP5, since endogenous AQP5 was also localized both at the APM and in vesicles (Figure 4C). Notably, a sub population of the YFP-AQP5 vesicles was found in close proximity of the TGN (Figures 4B,E). Although we could not determine whether these vesicles are endocytic or exocytic in nature, a time-lapse analysis revealed that they were stationary under basal conditions, possibly indicating that AQP5 is stored in an intracellular reservoir that may be mobilized during stimulation of water or protein secretion (Figure 4E), as shown for AQP2 in the kidney (Fushimi et al., 1997).

In summary PEI-mediated *in vivo* transfection is a powerful tool to rapidly and reliably express transgenes in rat salivary glands *in vivo*. Although this method is suitable to perform single cell imaging and immunocytochemistry, its main limitation is the low yield of transfection (1–2% of the cells of the parenchyma). Therefore, PEI is not suitable to carry out biochemical analysis or for gene therapy. Nonetheless, this approach provides the opportunity to perform localization and functional studies under physiological conditions using commercially available plasmids and in a relatively short time.

## Materials and methods

### Plasmids and cell lines

Human AQP5 and rat AQP5 were obtained from Dr. Bruce Baum (NIDCR, NIH) and subcloned in the following vectors pEGFP-C1, pEGFP-N1, pEYFP-C1, pEYFP-N1 (Clontech). TGN38-mCherry and YFP-TGN38 were a generous gift from Dr. Sarah Hamm-Alvarez (UCSC). GFP-actin, GFP-myosin Vc and GFP-TfnR were obtained from Dr. Julie Donaldson (NHLBI, NIH). GFP-Ribonuclease was a generous gift from Dr. David Yule (Rochester University). The 2X-EYFP vector was generated by inserting a copy of the YFP into a pEYFP-C and used to generate the 3X-EYFP using the same procedure. HSG-cell lines stably expressing YFP-AQP5 were prepared by lentiviral expression, as previously reported (Amornphimoltham et al., 2013).

### Cell cultures

HSG cells were cultured in Dulbecco's modified Eagle's medium/Ham's F-12 (1:1) (Invitrogen, CA) in the presence of 5% fetal bovine serum, 100 U/mL penicillin, and 100 mg/mL streptomycin (Sigma-Aldrich, MO) at 37°C in a 5% CO_2_ humidified atmosphere.

Growth factor-reduced matrigel (Trevigen, MD) was diluted in complete medium at the appropriate concentration (2.0–6.0 mg/mL), added to a 12-well tissue culture dish, and incubated for 1 h at 37°C before culturing of the cells. 50 cells/mm^2^ were added onto the pre-solidified matrigel and grown for different times. Acinar cells were recovered from the matrigel by incubating in cell recovery solution (Trevigen, MD) for 30 min on ice. Acinar cells released from the matrigel were spread on glass coverslips and dehydrated at 37°C for 15 min. Cells were then fixed in 2% formaldehyde/PBS, incubated first in blocking solution (10% FBS, 0.02% sodium azide in PBS) for 15 min, and later with various primary antibodies for 1 h at RT. Cells were then washed and incubated with the appropriate secondary antibodies for 1 h at RT. After 3 washes with blocking solution, nuclei were labeled by incubating the cells with Hoechst 33342 before mounting.

### Animal procedures

All the experiments were approved by the National Institute of Dental and Craniofacial Research (NIDCR, National Institute of Health, Bethesda, MD, USA) Animal Care and Use Committee. Sprague–Dawley male rats weighing 150–250 g were obtained from Harlan Laboratories Inc. (Frederick, MD) whereas the mice expressing cytoplasmic GFP were from Jackson laboratory (Masedunskas et al., 2011a). The animals were acclimated for 1 week before used for the procedures. Water and food were provided *ad libitum*. The animals were anesthetized by an IM injection of a mixture of Ketamine and Xylazine (100 mg/Kg an 20 mg/Kg respectively) with additional injections as needed.

### PEI-plasmid DNA complex

Polyethyleneimine (PEI) was purchased from Polyplus Transfection (New York, NY) as *in vivo*-jet PEI. Efficient transfection is achieved by using 12–24 μg of plasmid DNA/gland. Mix 50 μl of 10% glucose with the plasmid DNA and adjust the volume to 100 μl (solution 1). Mix 50 μl of 10% glucose with 7.5 μl of Jet PEI and adjust the volume to 100 μl (solution 2). Mix solution 1 and solution 2 and incubate them for 30 min at room temperature. Aspirate the transfection mixture with a syringe (30-gage needle) and make sure that no air bubbles are released when injecting the fluid.

### *In vivo* transfection

Anesthetized rats were secured into a previously described stereotactic device (Masedunskas et al., 2013) with the mandibles wide open and the cheeks extended to the sides. The tongue was folded toward the back of the mouth to expose the ductal orifices without obstructing the airways. The stereotactic device was at about 45 degrees and positioned under a stereo-microscope (SZX7, Olympus America, Center Valley, PA) to visualize the area below the tongue and locate the two orifices of the Wharton's ducts. A 30–40 cm PE-5 cannula (Strategic Applications, Libertyville, IL) was gently pushed into the orifice and introduced into the duct using bent sharp tweezers. To seal the cannula a small drop of histoacryl tissue glue was applied to the orifice. The cannula was connected to the syringe containing the transfection mixture that was injected e gradually over a 5 min time period by applying gentle pressure on the plunger. The cannula was removed from the mouth and the animal was allowed to recover. A warm environment was provided to facilitate the recovery and the animal was monitored for at least 2 h. The optimal yield of transfection was after 16–24 from the injection.

### Two-photon and confocal microscopy

Two-photon and confocal microscopy were performed on an IX81 inverted confocal microscope (Olympus, Melville, NY) modified to perform two-photon microscopy, as described previously (Masedunskas and Weigert, 2008). Intact and excised glands, and cultured cells, were imaged in the inverted setting by using the following objectives: UPLSAPO x10 NA 0.4, XLUMPFL20XW, 20X N.A. 0.95, and UPLSAPO 60X NA 1.2 (Olympus America Inc., Center Valley, PA). Intravital time-lapse imaging (Figure 4E) was performed as described in Masedunskas et al. (2011a) (acquisition speed 1 frame/sec).

### Immunofluorescence

The excised SGs were placed in optimum cutting temperature compound (Sakura Finetek USA Inc., Torrance, CA) and snap frozen in 2-methylbutane and liquid nitrogen. The glands were cut on silanated glass slides (10 μm thickness) and cryosections were incubated with blocking solution for 45 min followed by incubation with the appropriate primary antibody or with Texas-Red-Phalloidin (Invitrogen). Antibody against aquaporin 5 was purchased from Alomone labs.

### Conflict of interest statement

The authors declare that the research was conducted in the absence of any commercial or financial relationships that could be construed as a potential conflict of interest.
